# CRPGCN: predicting circRNA-disease associations using graph convolutional network based on heterogeneous network

**DOI:** 10.1186/s12859-021-04467-z

**Published:** 2021-11-12

**Authors:** Zhihao Ma, Zhufang Kuang, Lei Deng

**Affiliations:** 1 School of Computer and Information Engineering, Central South University of Forestry and Technology, Changsha, China; 2 School of Computer Science and Engineering, Central South University, Changsha, China

**Keywords:** CircRNA-disease, Graph convolutional network, Heterogenous network, Principal component analysis, Deep learning

## Abstract

**Background:**

The existing studies show that circRNAs can be used as a biomarker of diseases and play a prominent role in the treatment and diagnosis of diseases. However, the relationships between the vast majority of circRNAs and diseases are still unclear, and more experiments are needed to study the mechanism of circRNAs. Nowadays, some scholars use the attributes between circRNAs and diseases to study and predict their associations. Nonetheless, most of the existing experimental methods use less information about the attributes of circRNAs, which has a certain impact on the accuracy of the final prediction results. On the other hand, some scholars also apply experimental methods to predict the associations between circRNAs and diseases. But such methods are usually expensive and time-consuming. Based on the above shortcomings, follow-up research is needed to propose a more efficient calculation-based method to predict the associations between circRNAs and diseases.

**Results:**

In this study, a novel algorithm (method) is proposed, which is based on the Graph Convolutional Network (GCN) constructed with Random Walk with Restart (RWR) and Principal Component Analysis (PCA) to predict the associations between circRNAs and diseases (CRPGCN). In the construction of CRPGCN, the RWR algorithm is used to improve the similarity associations of the computed nodes with their neighbours. After that, the PCA method is used to dimensionality reduction and extract features, it makes the connection between circRNAs with higher similarity and diseases closer. Finally, The GCN algorithm is used to learn the features between circRNAs and diseases and calculate the final similarity scores, and the learning datas are constructed from the adjacency matrix, similarity matrix and feature matrix as a heterogeneous adjacency matrix and a heterogeneous feature matrix.

**Conclusions:**

After 2-fold cross-validation, 5-fold cross-validation and 10-fold cross-validation, the area under the ROC curve of the CRPGCN is 0.9490, 0.9720 and 0.9722, respectively. The CRPGCN method has a valuable effect in predict the associations between circRNAs and diseases.

## Background

With the advancement of science and technology, bioinformatics is increasingly at the forefront of scientific research. The relationships between diseases and drugs [1], the relationships between RNAs and diseases [2–4] are play an increasingly important role in the treatment and development of human diseases. Therefore, more and more scholars begin to invest in research in the direction of bioinformatics [5, 6]. Especially, circRNAs as non-coding RNA (ncRNAs), it has higher stability and integrity than other linear ncRNAs. Therefore, circRNAs can be used as a biomarker of diseases, it also has great potential in the treatment and diagnosis of diseases.

Although the formation and characteristics of circRNAs are basically discovered after a plenty of research by scientists, there are still dozens of biological functions that are still unclear. A large number of biologists prove the associations between circRNAs and diseases through experimental methods. Recently, some researchers point out that certain functions of ciRS-7 are related to human pathology and the development of cancer [7], its regulation of diseases and the mechanism in the development process and relate diseases are discovered by more studies. In addition, the functions of various other circRNAs are also being investigated. Usually, laboratory consumables are disposable, even some reusable equipment in the laboratory need manual maintenance. Therefore, as the number of experiments increases, such experiments base on experimental methods require a large deal of time and resources, resulting in high experimental costs. Consequently, it is more necessary to study the relationships between circRNAs and diseases based on computational methods.

Recently, an increasingly large number of researchers invest in research on the relationships between circRNAs and diseases based on computational methods. Lu et al. propose a method for the associations between circRNAs and diseases based on sequence and ontology representation, the k-mers is used to reduce dimensionality and the method apply Convolutional Neural Networks (CNN) to extract features, and then Long Short-Term Memory (LSTM) algorithm is used to feature learning [8]. Zhang et al. propose the PDC-PGWNNM method [9] approach to design circRNA-disease graph structure data using circRNA-miRNA interactions and miRNAs regulatory relationships in diseases, and the Weighted Nuclear Norm Minimization (WNNM) model is used to predict. Lei et al. propose the CDWBMS method [10], which uses a heterogeneous network to integrate the relationships between circRNAs and diseases, and it predicts the relationships between circRNAs and diseases based on an improved Weighted Biased Meta-Structure (WBMS) search algorithm. Wang et al. propose a algorithm based on Generative Adversarial Networks (GAN), which adopts the Extreme Learning Machine (ELM) classifier to predict [11]. Wei et al. propose a method called iCircDA-LTR [12], it utilize Learning to Rank (LTR) algorithm to rank the associations based on various predictive variables and characteristics in a supervised manner.

In addition to the above studies, The Graph Convolutional Network (GCN) [13], The Random Walk with Restart (RWR) [14] and The Principal Component Analysis (PCA) [15] have also played an indelible role. Jin et al. propose NIMCGCN method to predict miRNA-disease associations establish on Neural Inductive Matrix Completion (NIMC) with GCN [16]. Wang et al. propose a calculation method is referred to GCNCDA [17] based on Fast learning with Graph Convolutional Networks (FastGCN) combine with Forest by Penalizing Attributes (Forest PA) classifier to predict potential circRNA-disease associations. Pan et al. propose an updated predictor DimiG 2.0 [18], which uses a semi-supervised multi-label GCN to infer the relationships between miRNAs and diseases on the interaction network between Protein-coding genes (PCGs) and miRNAs.

RWR can captures the multifaceted relationships between circRNAs or between diseases and treats the circRNAs matrix or diseases matrix as a graph structure, and RWR is utilised to capture information about the overall structure of the graph. Such as RWRKNN [19], IIRWR [20], TRWR-MB [21], MRWMDA [22]. In this paper, the RWR algorithm is used to calculate the similarity between circRNAs and the similarity between diseases in preparation for the subsequent PCA feature extraction.

In numerous different directions of research [23, 24], PCA played an important role. The circRNAs and diseases in this paper have a host of different attributes. If these datas are analyzed separately, their information may not be fully utilized, and some datas will be isolated. This kind of datas use leads to results that are subject to varying degrees of bias. Therefore, the PCA algorithm is required to perform a comprehensive analysis of the original data while also perform data dimensionality reduction.

Based on the discretion and research of the above methods, a novel and reliable method is proposed in this paper, which is based on Graph Convolutional Networks (GCN) to predict the associations between circRNAs and diseases, called CRPGCN. Compared to other algorithms, such as the GCNCDA, it uses the GCN algorithm as a feature extraction method and uses Forest PA classifier to classify features, but it does not consider neighbour nodes associations. In contrast, CRPGCN maximises the performance of GCN by first extracting features and noise reduction from the associations between circRNAs and diseases, and then performing feature learning that takes into account the associations between neighbouring nodes. Furthermore, in the comparative experiments in this paper, it can also be seen that the CRPGCN method outperforms some advanced GCN methods.

The main contributions of this work are summarized as follows:

- The CRPGCN method incorporates the RWR similarity calculation method and the PCA feature extraction method, allowing the calculated nodes to better combine the similarity between neighbouring nodes while greatly reducing the impact on the prediction results.
- The CRPGCN algorithm improves prediction accuracy and has the highest AUC values and AUPR values when compared to advanced algorithms.
- The GCRGCN algorithm is more stable than some of the advanced algorithms, and its AUCs are stable when compared by a variety of methods with different datasets.
- By comparing various evaluation metrics, the CRPGCN algorithm outperforms other advanced algorithms in terms of overall performance.

## Benchmark datasets

The selection of dataset is one of the keys to study and predict circRNA-disease associations. The gene-based circRNA similarity is the basis for the composition of the comprehensive similarity matrix in this paper, and it makes an important contribution in the study by Ding et al. [25]. Meanwhile, circR2Disease [26] can be used to construct gene-based circRNA similarity based on the study by Hang et al. [27]. In summary, circR2Disease dataset is used as the benchmark to calculate circRNA-disease associations matrix A, gene-based circRNA similarity CGS. circRNA GIP kernel similarity CIS and disease GIP kernel similarity DIS are thereafter calculated using A. circBase [28] is considered as the benchmark database, which combines the CGR algorithm to calculate the sequence-based similarity of each circRNA pair. In addition, the DAG information from the MeSH database provided the basis for calculating the semantic similarity between diseases.

## Methods

**Fig. 1:**
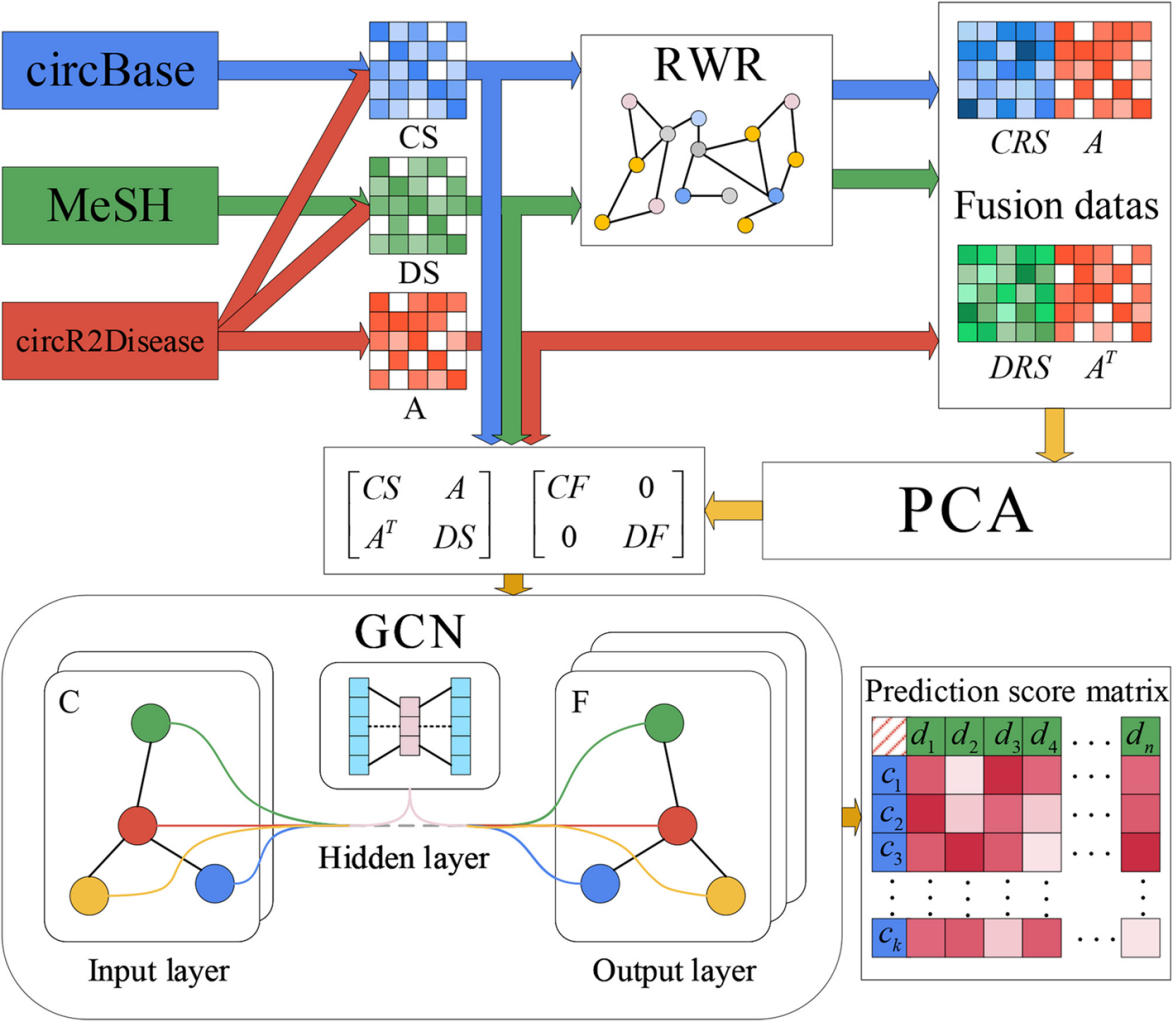
The flowchart of CRPGCN

In this paper, a novel algorithm is proposed, which is called CRPGCN, show as Fig. 1. In this study, the dataset needed to be preprocessed to construct adjacency matrices and feature matrices connecting circRNAs to diseases by the following methods:

The adjacency matrix A is obtained from the known circRNA-disease associations in the dataset. The circRNA comprehensive similarity matrix CS consists of the circRNA GIP kernel similarity matrix CIS, the circRNA gene-based similarity matrix CGS and the circRNA sequence-based similarity matrix CES. Thereafter, the disease comprehensive similarity matrix DS is composed of the disease GIP kernel similarity matrix DIS and the disease semantic similarity matrix DSS. Thereafter, the CRPGCN method is trained by constructing heterogeneous adjacency matrices and heterogeneous feature matrices from A, CS and DS obtained in the above manner. The CRPGCN algorithm flow is as follows: Step 1: The matrices A, CS and DS given by data pre-processing are fed into the CRPGCN. Step 2: The RWR algorithm is used to aggregate the CS matrix and DS neighbour node information respectively to obtain the CRS and DRS. Step 3: The CRS matrix and DRS matrix are combined with the adjacency matrix A respectively, and the PCA is used to reduce the dimension and extract the features to obtain the feature matrices CF and DF separately. Step 4: The CS, DS and A are used to form the heterogeneous adjacency matrix ${\mathrm{A_{cd}}}$, after which CF and DF are used to compose the heterogeneous feature matrix CD, and finally the GCN algorithm is used for feature learning and scores calculation between circRNAs and diseases. The relationships between circRNAs and diseases is treated as graph-structured data by CRPGCN, which makes full use of the associations between each node and its neighbours to learn informations about similar nodes, while isolated nodes can also be well handled. Ultimately, the accuracy and stability of the CRPGCN algorithm is demonstrated by comparative experiments. In particular, the above steps will be described in detail in the following section.

### Construct circRNA-disease adjacency matrix

The establishment of the adjacency matrix A (see Additional file 1) uses the known association relationships between circRNAs and diseases in the CircR2Disease dataset. A(i,j) is set to 1 when there is an associations between circRNAs and diseases, otherwise it is set to 0, is given by the following:

1 $$\begin{aligned} {\mathrm{A}}(i, j)=\left\{ \begin{array}{ll} 1 &\, \ \ \ \ c_{i} \,\, {\mathrm{and} } \,\, {d}_{j} \,\, {{\mathrm{has}} \,\, {\mathrm{related}} } \\ 0 &\, \ \ \ \ \, {\mathrm{otherwise}} \end{array}\right. \end{aligned}$$

### Construct circRNA GIP kernel similarity

For a circRNA $c_i$, IP$_1 (c_i)$ value is defined as the *i*-*th* row of the circRNA-disease associations matrix A. The calculation method for the GIP kernel similarity between each pair of $c_i$ and $c_j$ is shown as:

2 $${\text{CIS}}\left( {c_{i} ,c_{j} } \right) = \exp \left( { - \gamma _{c} \left\| {{\text{IP}}_{1} \left( {c_{i} } \right) - {\text{IP}}_{1} \left( {c_{j} } \right)} \right\|^{2} } \right)$$

3 $$\begin{aligned}&\gamma _{c}=\gamma _{c}^{\prime } /\left( \frac{1}{n} \sum _{i=1}^{n}\left\| {\mathrm{I P}}_{1}\left( c_{i}\right) \right\| ^{2}\right) \end{aligned}$$

where CIS represents the GIP kernel similarity of $c_i$ and $c_j$. $\gamma _c$ is used to control the bandwidth, it represents the regularized Gaussian interaction attribute kernel similarity bandwidth based on the new bandwidth parameter $\gamma _{m}^{\prime }$. $\gamma _{m}^{\prime }$ is set to 1. *n* represents the number of circRNA. The disease GIP kernel similarity DIS is calculated in the same way.

### Construct gene-based circRNA similarity

Because similar RNAs tend to regulate similar genes, genes have been widely used to infer RNA similarity. In this study, to construct the gene-based circRNA similarity, the circRNA-gene associations adjacency matrix ${\mathrm{A}}_{\mathrm{cg}}$ must be constructed first. Where ${\mathrm{A}}_{\mathrm{cg}}$ is set to 1 to indicate that $g_i$ and $g_j$ are related, otherwise it is set to 0. Similar to the circRNA GIP kernel similarity calculation method, the GIP kernel similarity matrix GIS of the gene is constructed. The gene-based circRNA similarity matrix CGS is constructed [27] through the ${\mathrm{A}}_{\mathrm{cg}}$ and GIS matrix, it is given by:

4 $$\begin{aligned} \text {CGS}={\mathrm{A}}_{\mathrm{cg}} \times \text {GIS} \times {\mathrm{A}}_{\mathrm{c g}}^{\mathrm{T}} \end{aligned}$$

where ${\mathrm{A}}_{\mathrm{cg}}^{\mathrm{T}}$ is the transpose of ${\mathrm{A}}_{\mathrm{cg}}$.

### Construct sequence-based circRNA similarity

The method rest on Chaos Game Representation (CGR) [29] can transform circRNA sequences into the corresponding spectral format. This method can exploit CGR coordinates to convert circRNA sequences into CGR radian sequences.

This method uses the Pearson correlation coefficient to quantify the similarity and difference between the position information and the nonlinear information for calculates the sequence-based circRNAs similarity matrix CES. By combining the method of Zheng et al. the CGR space [30] is first divided into $8\times 8$ grids and the *i*-*th* grid can be expressed as:

5 $$\begin{aligned} {\mathrm{grid}}_{\mathrm{i}}=\left( X_{i}, Y_{i}, Z_{i}\right) \end{aligned}$$

Furthermore, the quantified position information $X_i$ and $Y_i$ of $grid_i$ is obtained by accumulating the horizontal coordinate value $x_j$ and vertical coordinate value $y_j$ in each grid respectively, which can be presented as follows:

6 $$\begin{aligned} \left\{ \begin{array}{l} X_{i}=\sum _{j=1}^{{Num}_{i}} {\mathrm {x}}_{j} \quad {\mathrm{if}}\, {\mathrm{point }}\,\left( {\mathrm {x}}_{j}, {\mathrm {y}}_{j}\right) \,{ {\mathrm {in}} }\, \text {grid}_{\mathrm{i}} \\ \\ Y_{i}=\sum _{j=1}^{{Num}_{i}} {\mathrm {y}}_{j} \quad { {\mathrm {if}} } \,{\mathrm{point}}\, \left( {\mathrm {x}}_{j}, {\mathrm {y}}_{j}\right) \, { {\mathrm {in}} }\, \text {grid}_{\mathrm{i}} \end{array}\right. \end{aligned}$$

where $Num_i$ denotes the number of points in the *i*-*th* $grid_i$, $X_i$ denotes the sum of the horizontal coordinate values $x_j$ for all points in the *i*-*th* $grid_i$, and $Y_i$ denotes the sum of the horizontal coordinate values $y_j$ for all points in the *i*-*th* $grid_i$. $Z_i$ is used to represent the *z*-*score* of each grid to quantify the non-linear information, which is calculated as:

7 $$\begin{aligned} Z_{i}=\frac{N u m_{i}-\frac{\sum _{k=1}^{N_{g}} N u m_{k}}{N_{g}}}{\sqrt{\frac{1}{N_{g}} \sum _{h=1}^{N_{g}}\left( N u m_{h}-\frac{\sum _{f=1}^{N_{g}} N u m_{f}}{N_{g}}\right) ^{2}}} \end{aligned}$$

where $N_g$ is 64, which means the total number of grids.

Finally, based on the above calculation of the $X_i$ , $Y_i$ and $Z_i$ attributes of each $grid_i$, the following equation is fused to construct a description array $des(c_i)$ for all grids of the circRNA sequence being calculated:

8 $$\begin{aligned} {\mathrm{desc }}\left( c_{i}\right) ={ \left( \text {grid}_{1}, \text { grid }_{2}, \ldots , \text { grid }_{{\mathrm{N}}_{\mathrm{g}}}\right) } \end{aligned}$$

Then, the Pearson correlation coefficient is used to determine the sequence similarity CES, it can be presented as follows:

9 $$\begin{aligned} {\mathrm{CES}}\left( c_{i}, c_{j}\right) =\frac{{\mathrm{Cov}}\left( {\mathrm{desc }}\left( c_{i}\right) , {\mathrm{desc }}\left( c_{j}\right) \right) }{{\mathrm{D}}\left( {\mathrm{desc }}\left( c_{i}\right) \right) \times {\mathrm{D}}\left( {\mathrm{desc }}\left( c_{j}\right) \right) } \end{aligned}$$

where Cov($*$) represents the covariance, D($*$) represents the variance, $c_i$ represents the *i*-*th* circRNA.

### Constructing disease semantic similarity

The DAG associations between diseases can help to calculate the similarity between each pair of diseases. The more DAG correlations between two diseases, the greater their similarity. The contribution value of the diseases can quantify the DAG correlation between the two diseases. Calculation of diseases contribution values based on the MeSH dataset, which is given by:

10 $$\begin{aligned} {\mathrm{S}}\left( d_{i}, f\right) =\log \left( 1+\frac{ \text{ the } \text{ number } \text{ of } \text {DAGs including } f}{\text {the number of disease }}\right) \end{aligned}$$

Through the contribution value of the diseases, the semantic similarity between the diseases is calculated, DSS is described as follows:

11 $$\begin{aligned} {\text {DSS}}\left( d_{i}, d_{j}\right) =\frac{\sum _{t \in {\mathrm{T}}\left( d_{i}\right) \cap \left( d_{j}\right) }\left( {\mathrm{S}}\left( d_{i}, f\right) +{\mathrm{S}}\left( d_{j}, f\right) \right) }{\sum _{t \in {\mathrm{T}}\left( d_{i}\right) } {\mathrm{S}}\left( d_{i}, f\right) +\sum _{t \in {\mathrm{T}}\left( d_{j}\right) } {\mathrm{S}}\left( d_{j}, f\right) } \end{aligned}$$

where T$(d_i)\cap$T$(d_j)$ represents the set of common ancestor nodes of the two diseases $d_i$ and $d_j$.

### Data fusion

The circRNA comprehensive similarity matrix CS is obtained by fusing the matrices CIS, CGS and CES. If the gene-based circRNA similarity is not 0, the average value of CIS, CGS and CES is united as the current circRNA comprehensive similarity CS (see Additional file 2). Otherwise, the average value of CIS and CGS is used as the CS of circRNA. The comprehensive similarity CS is given by:

12 $$\begin{aligned} \text {CS}=\left\{ \begin{array}{ll} \frac{\text {CIS}\left( c_{i}, c_{j}\right) +\text {CGS}\left( c_{i}, c_{j}\right) +\text {CES}\left( c_{i}, c_{j}\right) }{3} &\, {\mathrm{if}}\, \text {CES}\ne 0 \\ \\ \frac{\text {CIS}\left( c_{i}, c_{j}\right) +\text {CGS}\left( c_{i}, c_{j}\right) }{2} &\, {\mathrm{if}} \, \text {CES}=0 \end{array}\right. \end{aligned}$$

If the diseases has no DAG associations, certain semantic similarities cannot be calculated. By analyzing disease similarity measures from multifaceted, in order to calculate the similarity between diseases more comprehensively, DIS and DSS are needed to be fused together. The disease comprehensive similarity DS (see Additional file 3) between diseases $d_i$ and $d_j$ is defined as follows:

13 $$\begin{aligned} \text {DS}=\left\{ \begin{array}{ll} \frac{\text {DIS}\left( d_{i}, d_{j}\right) +\text {DSS}\left( d_{i}, d_{j}\right) }{2} &\, {\mathrm{DAG}} \text { association } \text {exists} \\ \\ {\mathrm{DIS}}\left( d_{i}, d_{j}\right) &\, {\mathrm{otherwise}} \end{array}\right. \end{aligned}$$

### CRPGCN algorithm

In this section, the implementation of the CRPGCN algorithm is described in detail. The adjacency matrix A, circRNA comprehensive similarity matrix CS and disease comprehensive similarity matrix DS are used as the input datas for CRPGCN, and the output is the score matrix. The specific process is shown in Algorithm 1.

From lines 1–7 of the CRPGCN algorithm, CS and DS are used by the RWR algorithm to fuse the similarity information of neighbouring nodes to obtain CRS and DRS. Because the similarity relationships between each node and its neighbours has an important influence on the prediction result, the RWR algorithm can combine well to calculate the relationships between nodes and their neighbours. RWR combines the similarity [31] between neighbouring nodes by random walk and adjusts the degree of integration of the combined neighbouring nodes by edge weights. The calculation method [19] of RWR is defined as:

14 $$\begin{aligned} \vec {r}_{l}=c {\widetilde{W}} \vec {r}_{l}+(1-c) \vec {e}_{l} \end{aligned}$$

where $W=[w_{i,j}]$ is the transfer probability matrix and ${\widetilde{W}}$ is the matrix after normalisation of *W*. $\vec {e}_{l}$ is the initial vector of $k \times 1$ and is the row vector of the CRS or DRS. *c* is the restart probability. Based on subsequent experiments *c* is set to 0.4. $\vec {r}_{l}$ is the similarity vector obtained after the RWR calculation.

With the RWR algorithm, there is a certain probability that the walk process of the computed nodes will combine the similarity between the lowly associated neighbouring nodes, and the generation of similarity noise is inevitable. In order to reduce the impact of similarity noise on the computation results, the PCA algorithm is invoked. In rows 9–21, by using the PCA algorithm to extract features while noise reduction of the similarity matrix, the final obtained feature matrices CF, DF can be better learned by GCN, the calculation [32] of the feature matrix is shown below: 
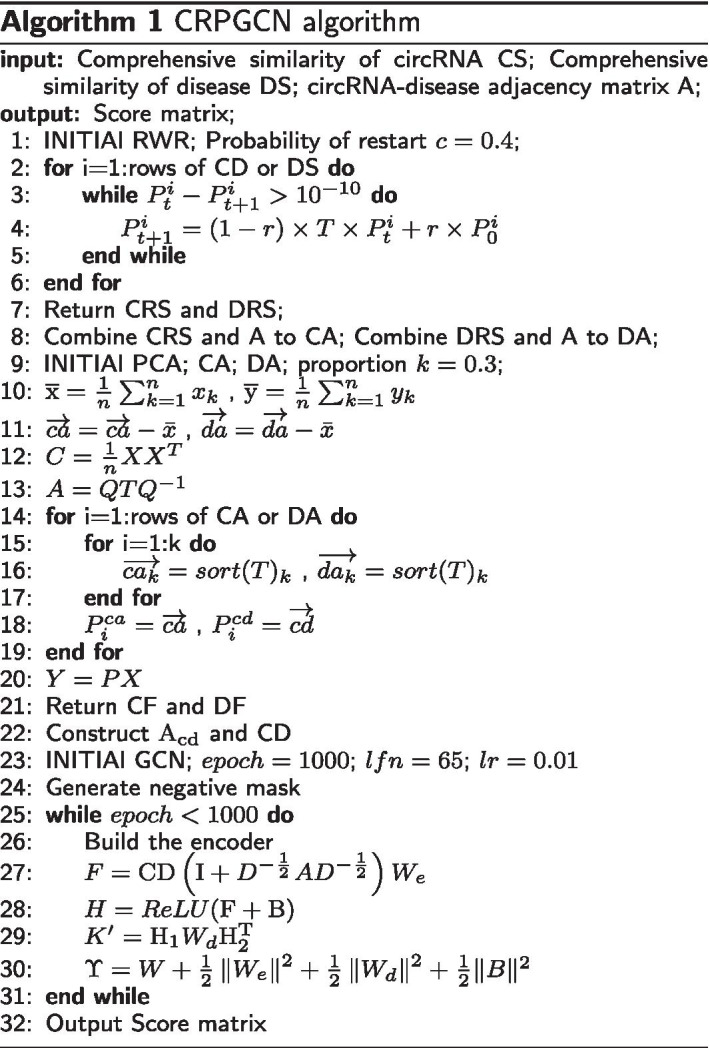

15 $$\begin{aligned} {\mathrm{Y}}=PX \end{aligned}$$

In line 22, By using noise reduction on the similarity matrix, the final result is used as the feature matrices CF (see Additional file 4), DF (see Additional file 5) for circRNAs and diseases. At the same time, the noise reduction matrix is not enough for the GCN method to find the associations between nodes more easily [33], the concept of heterogeneous adjacency matrix and heterogeneous feature matrix are introduced for better feature embedding. Their construction methods are shown as follows:

16 $$\begin{aligned} {\mathrm{A}}_{\mathrm{c d}}= & \, \left[ \begin{array}{cc} {\mathrm{C S}} &\, {\mathrm{A}} \\ {\mathrm{A}}^{\mathrm{T}} &\, {\mathrm{D S}} \end{array}\right] \end{aligned}$$

17 $$\begin{aligned} {\mathrm{CD}}= & \, \left[ \begin{array}{cc} {\mathrm{C F}} &\, {\mathrm{0}} \\ {\mathrm{0}} &\, {\mathrm{D F}} \end{array}\right] \end{aligned}$$

The learning method of the GCN is defined specifically from lines 23 to 32. According to the definition of GCN, the formula for the convolution of the adjacency matrix A_cd_ with the identity matrix CD is given by:

18 $$\begin{aligned} {\mathrm{F}}={\mathrm{C D}}\left( {\mathrm{I}}+D^{-\frac{1}{2}} A D^{-\frac{1}{2}}\right) W_{e} \end{aligned}$$

where the Fourier series matrix $W_e$ is the training weight matrix, then ${\mathrm{C D}}\left( {\mathrm{I}}+D^{-\frac{1}{2}} A D^{-\frac{1}{2}}\right)$ represents the hidden associations between circRNAs or diseases nodes and potential factors. It can be converted into a hidden matrix H through the $W_e$. I is the identity matrix. By introducing the deviation matrix B into the hidden matrix H through the activation function. The initialisation [34] of the trainable matrices $W_e$, $W_d$ and *B* is provided by Glorot et al. as follows:

19 $$\begin{aligned} \Upsilon= & \, W + \frac{1}{2}\left\| W_{e}\right\| ^{2} +\frac{1}{2}\left\| W_{d}\right\| ^{2}+\frac{1}{2}\Vert B\Vert ^{2} \end{aligned}$$

20 $$\begin{aligned} W= & \, \sqrt{\frac{\sum _{i j ; \Phi _{p, j}=1 o r \Phi _{n, j}=1}\left( M_{i j}^{\prime }-M_{i j}\right) }{\sum _{i j}\left( \Phi _{p, i j}+\Phi _{n, i j}\right) }} \end{aligned}$$

where $\Phi _{p}$ and $\Phi _{n}$ are randomly selected positive and negative samples for this experiment. *W* is used to minimise the prediction error during the iterative process, and it is calculated as shown in Eq. (19). The constraints on the weight matrices in the encoder and decoder are defined by the remaining three terms separately. Because the ratio of positive and negative samples affects the experimental training results, this experiment validates the optimal ratio of positive and negative samples, and the validation results and discussion will be given in the next section.

## Results

### Evaluation method and metrices

The ROC curve is drawn based on *TPR* and *FPR*. The calculation method of *TPR* is as follows:

21 $$\begin{aligned} T P R=\frac{T P}{T P+F N} \end{aligned}$$

where *TPR* represents the percentage of all samples that are actually positive that are correctly judged as positive. In addition, *FPR* is calculated as follows:

22 $$\begin{aligned} FPR=\frac{FP}{FP+TN} \end{aligned}$$

where *FPR* is the percentage of all samples that are actually negative that are incorrectly judged to be positive.

The experiment used a variety of methods to assess performance, including recall (Recall), F1 score (F1), accuracy (ACC), Matthew correlation coefficient (MCC), area under the receiver operating characteristic curve (AUC) and area under precision-recall curve (AUPR). They are defined as:

23 $$\begin{aligned} F 1= & \, \frac{2 \times T P}{2 T P+F P+F N} \end{aligned}$$

24 $$\begin{aligned} M C C= & \, \frac{T P \times T N-F P \times F N}{\sqrt{(T P+F P)(T P+F N)(T N+F P)(T N+F N)}} \end{aligned}$$

25 $$\begin{aligned} ACC= & \, \frac{T P+T N}{T P+T N+F P+F N} \end{aligned}$$

26 $$\begin{aligned} Recall= & \, \frac{T P}{T P+F N} \end{aligned}$$

where *TP* is true positive, indicating the number of positive samples that are correctly classified, and *FN* is false negative, indicating the number of negative samples that are incorrectly classified. *FP* is false positive, which means the number of positive samples that are incorrectly classified as negative; *TN* is true negative, which means the number of negative samples that are correctly classified.

**Fig. 2:**
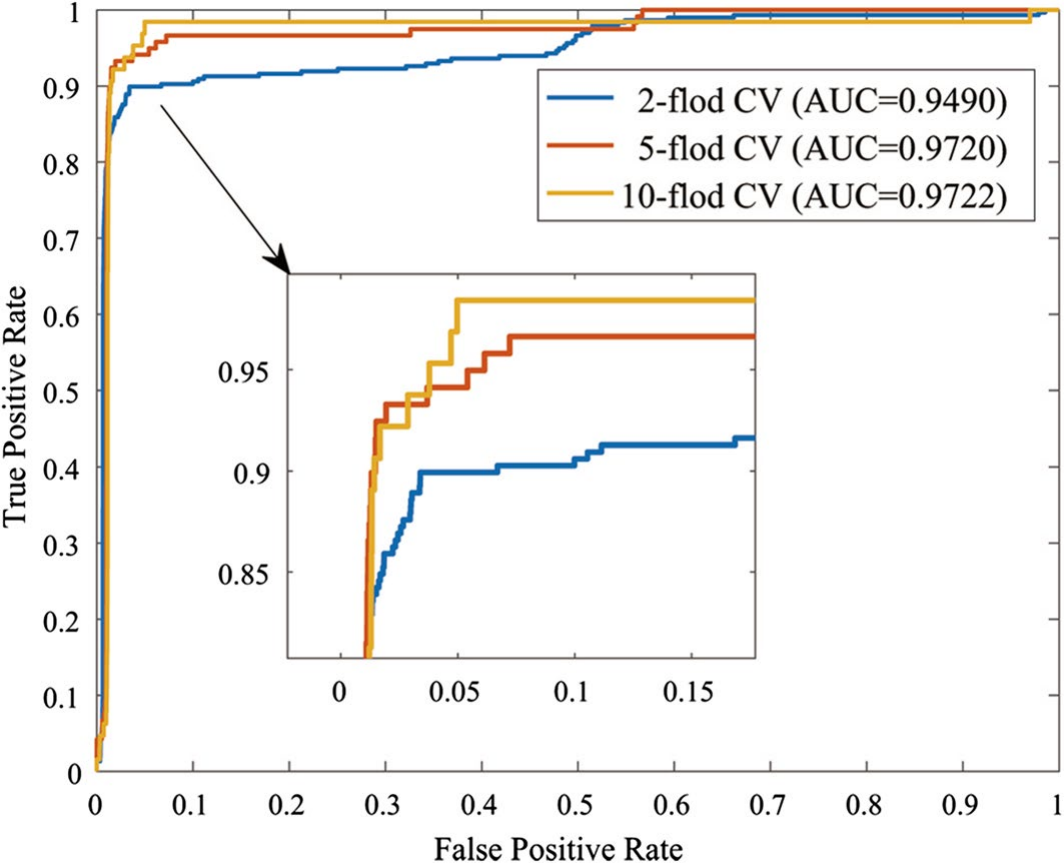
The ROC curves of the CPRGCN with k-fold corss-validation

### k-fold cross validation

In this section, k-fold cross-validation (CV) is used to assess the performance of CRPGCN. The dataset used for this experiment is derived from a combined dataset of 533 circRNAs associated with 89 diseases obtained by screening the circBase, circR2Disease and MeSH databases. In order to assess the performance of CRPGCN more accurately, the dataset is randomly sampled. According to the AM matrix, when the AM matrix is 1, it is a positive sample, otherwise it is a negative sample, after which the positive sample is randomly disrupted while it is divided into 5 equal parts, then the negative sample data is taken 5 times the positive sample, and finally the positive and negative samples are combined as training samples. In addition to the associations between circRNAs and diseases in the dataset itself, the potential associations between circRNAs and diseases also has a significant impact on the final results, and the lantent factor number (LFN) parameter is adjusted to the optimal value, which is presented in the next section. In addition, the ratio of positive to negative samples also plays a crucial role in the outcome of the experiment. The ROC curves are shown in Fig. 2, with the final AUC values of 0.9490, 0.9720 and 0.9722 for the 2-fold CV, 5-fold CV and 10-fold CV respectively.

**Fig. 3:**
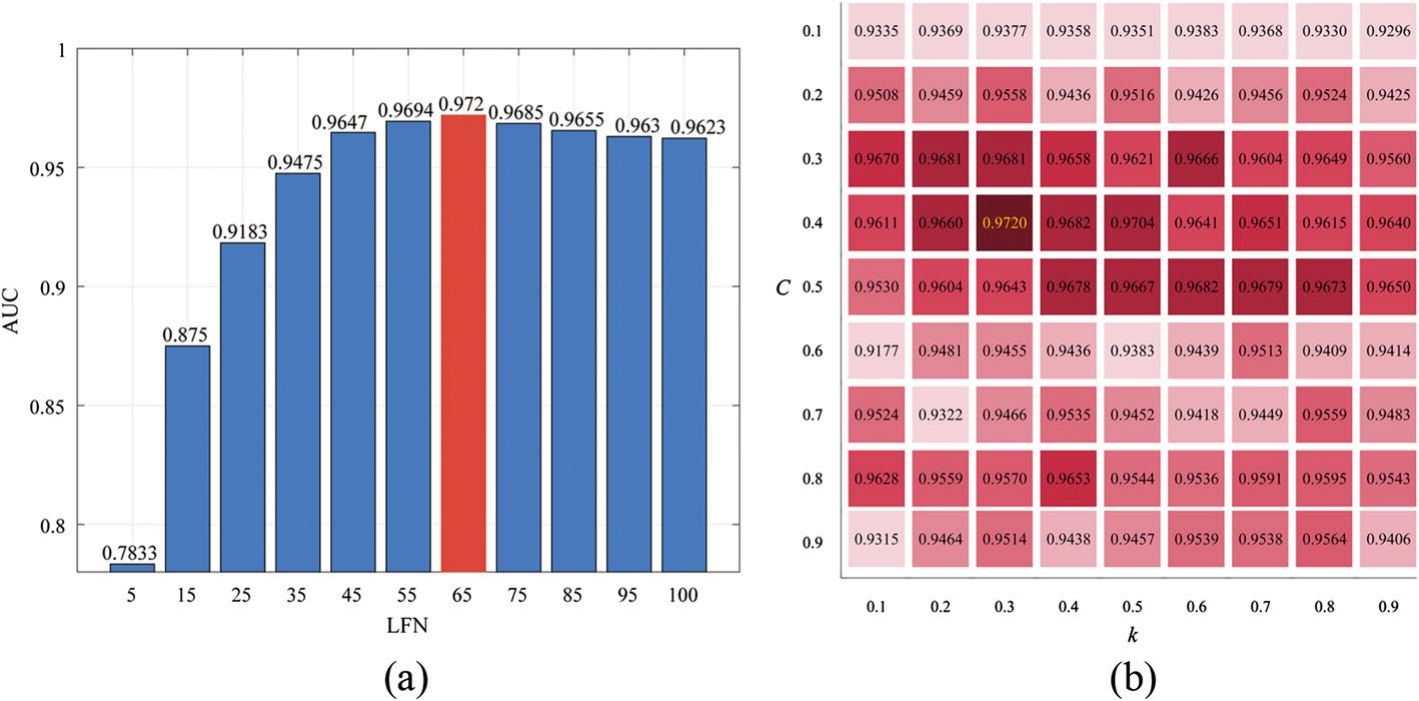
Analysis of parameters **a** Compare the AUC values with different *c* and *k*, **b** compare the AUC values with different lantent factor number

### Analysis of parameters

The key parameters of the CRPGCN algorithm have a huge impact on the results [35], thus in this section, the three primary parameters will be analysed.

**Table 1:** Compare the AUC values with different LFN

LFN	Twofold CV	Fivefold CV	Tenfold CV
5	0.7705	0.7833	0.7882
15	0.8548	0.8750	0.8820
25	0.8950	0.9183	0.9247
35	0.9321	0.9475	0.9500
45	0.9352	0.9647	0.9573
55	0.9434	0.9694	0.9641
65	0.9490	0.9720	0.9722
75	0.9442	0.9685	0.9647
85	0.9434	0.9655	0.9628
95	0.9401	0.9630	0.9675
100	0.9401	0.9623	0.9612

**Fig. 4:**
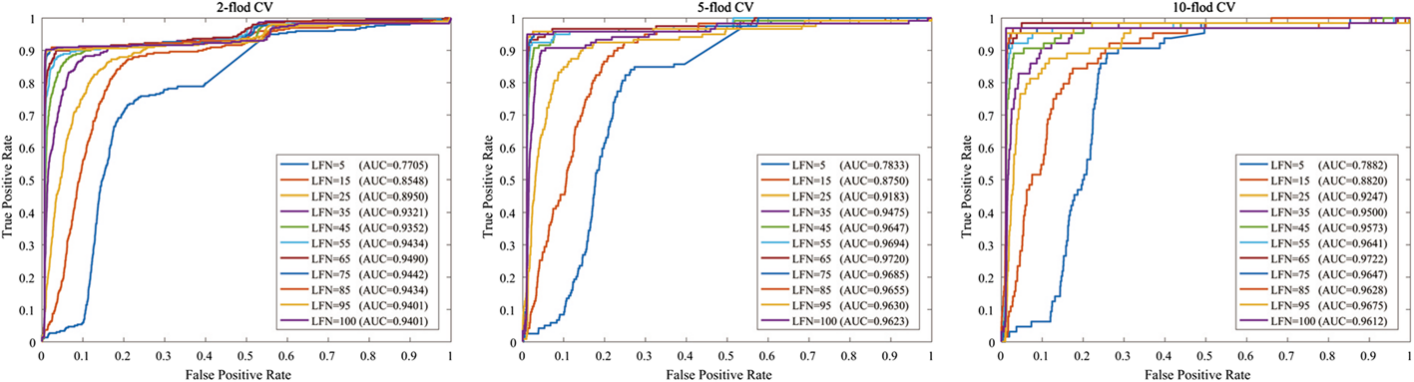
Comparison of ROC curves for different LFN under k-fold CV

In the CRPGCN, the LFN is one of the foundations on which it is constructed, and it plays an integral role in this experiment. Therefore, this subsection evaluates the impact on the CRPGCN algorithm based on the variation of LFN, which is set to range from 5 to 100 and validated by AUC values. In addition to this, a fivefold CV of the dataset is performed by fixing the optimal values of the remaining parameters constant. As shown in the histogram in Fig. 3a, the trend of the AUC value is monotonically increasing as the LFN goes from 5 to 65. From 65 to 100 there is a monotonically decreasing pattern. In addition, the best AUC value of 0.9720 is obtained at an LFN of 65. By adjusting the LFN to a reasonable value, the associations between circRNAs and diseases can be strengthened, thus making the prediction more accurate.

**Fig. 5:**
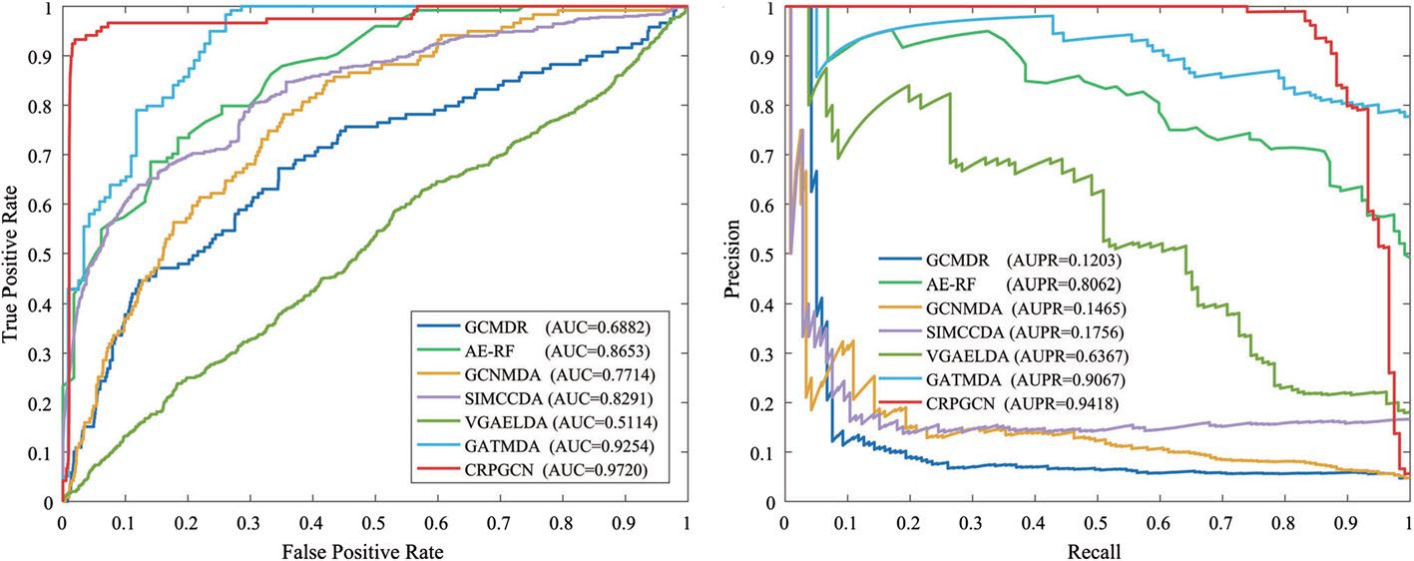
The ROC and P–R curves of different models under 5-fold cross-validation

In addition, the restart probability *c* of the RWR and the proportion *k* of the truncated vector of the PCA also have a large impact on the AUC of the CRPGCN. *c* means the probability of the computed node returning to the original node in the next step, and 1-*c* is the probability of being computed to reach a neighbouring node. *k* represents the number of matrix columns of length *k* of the matrix selected by the PCA processing matrix as the columns of the feature matrix. Because the distributions of both *c* and *k* are between 0 and 1, the experiments in this section set their step sizes to 0.1. From the results, it is shown that when *c* is between [0.3,0.5] and *k* is between [0.1,0.9], the average AUC values are 0.9643, 0.9658, and 0.9645, respectively. when *c* is 0.5 and *k* is between [0.4,0.8], the AUC values reached one of the peaks, with a range average AUC value of 0.9676, but they did not reach the highest value. The best AUC value is 0.9720 when *c*=0.4 and *k*=0.3. The experimental validation shows that although there are some outstanding AUC values in different ranges, the highest AUC values can only be obtained by setting the values of *c* and *k* reasonably, and the results are shown in Fig. 3b.

**Fig. 6:**
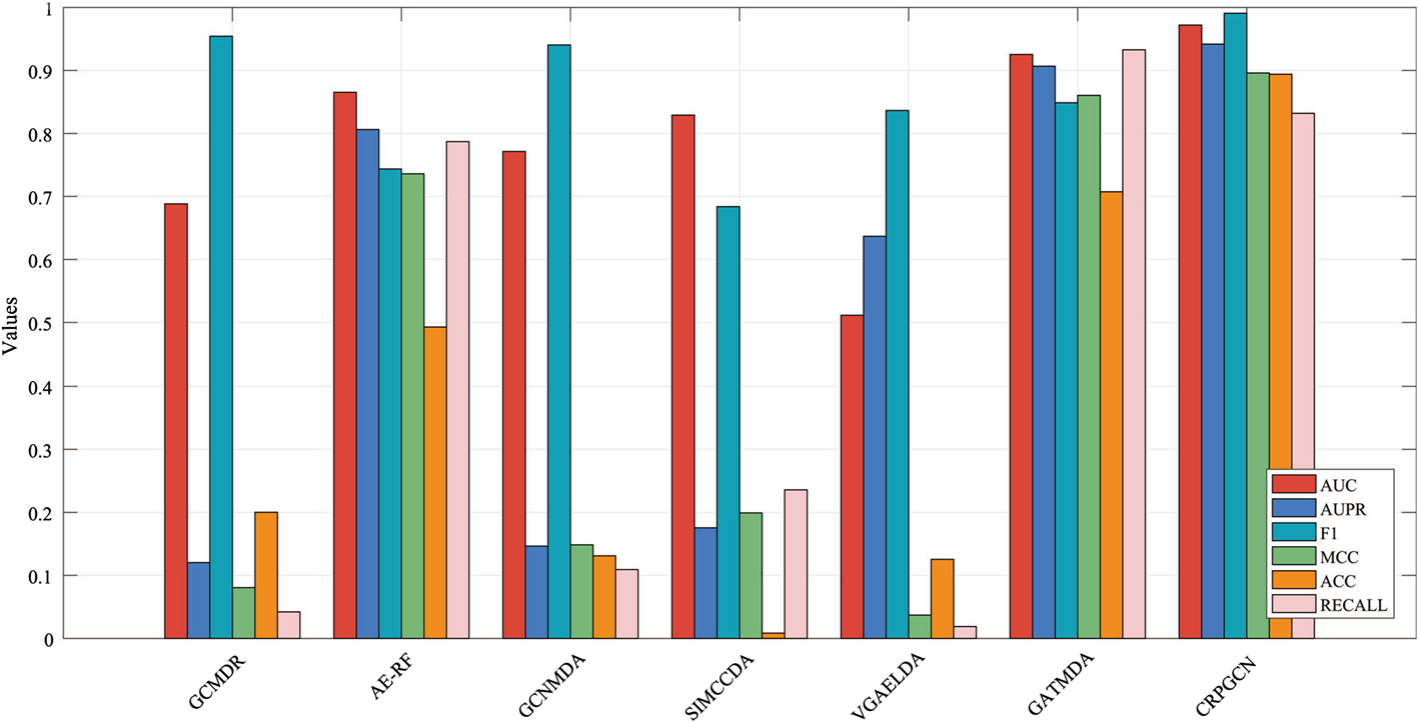
Comparison with multiple evaluation metrics

To further demonstrate the validity of the parameters, the results of the experiments at twofold CV, fivefold CV and tenfold CV of different LFN will be presented here, and the results prove the conclusions in the CRPGCN article to be correct. As shown in Fig. 4 and Table 1 (Tables 2, 3).

**Table 2:** Details of four datasets

DATASET	circRNAs	Diseases	Associations
DataSet-1	330	48	354
DataSet-2	661	100	736
DataSet-3	512	71	609
DataSet-4	533	89	612

**Table 3:** Compare the AUC values with different models

DATASET	CRPGCN	CRPGCN-I	CRPGCN-II
DataSet-1	0.9554	0.9335	0.6686
DataSet-2	0.9512	0.9458	0.5681
DataSet-3	0.9461	0.7441	0.6347
DataSet-4	0.9720	0.7552	0.6097

**Fig. 7:**
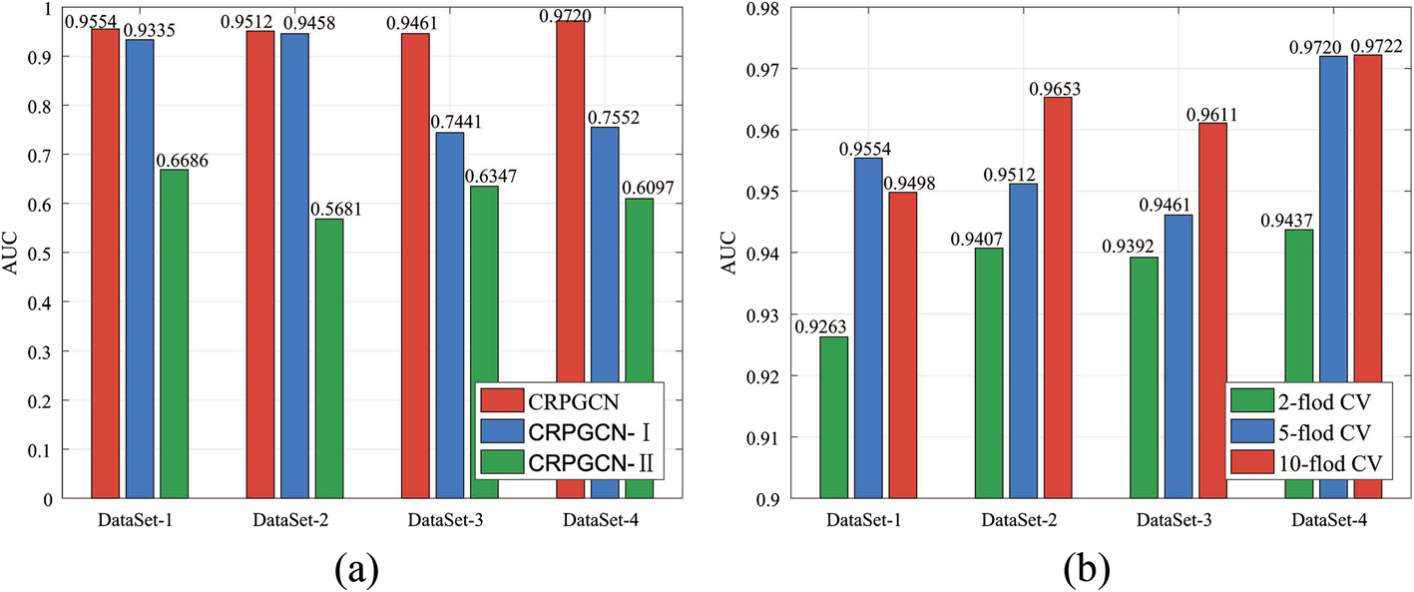
Comparison of the three algorithm modes of 4 different datasets under GRPGCN. **a** CRPGCN is the method proposed in this paper, which undergoes RWR for similarity aggregation and PCA feature extraction. The CRPGCN-I method is a direct fusion for feature extraction by PCA algorithm after comprehensive similarity calculation; CRPGCN-II is a direct fusion after comprehensive similarity calculation, without RWR for similarity aggregation and PCA feature extraction. **b** Represents the AUC values of twofold CV, fivefold CV and tenfold CV of the three models under four datasets

**Table 4:** Comparison with multiple evaluation metrics

DATASET	AUC	AUPR	F1	MCC	ACC	RECALL
GCMDR	0.6882	0.1203	0.9543	0.0806	0.2002	0.0420
AE-RF	0.8653	0.8062	0.7436	0.7359	0.4928	0.7870
GCNMDA	0.7714	0.1465	0.9403	0.1485	0.1311	0.1092
SIMCCDA	0.8291	0.1756	0.6839	0.1992	0.0083	0.2358
VGAELDA	0.5114	0.6367	0.8364	0.0370	0.1255	0.0188
GATMDA	0.9254	0.9067	0.8487	0.8604	0.7075	**0.9327**
CRPGCN	**0.9720**	**0.9418**	**0.9907**	**0.8959**	**0.8940**	0.8319

Bold indicates the Area Under the receiver operating characteristic Curve (AUC) is plot by TPR and FPR, and the Area Under Precision-Recall curve (AUPR) is plot by Recall and Precision. Precision = TP/(TP + FN)

### Comparison with existing methods

In order to verify the reliability of the algorithm, CRPGCN algorithms is used in this experiment to compare it with other excellent prediction method. As shown in Fig. 5. The GCMDR [36] is developed by Huang et al. to predict the relationships between miRNAs and drugs, and GCN to be used by it for extraction feature and final scores calculation. The AE-RF [37] is developed by K. Deepthi et al. to predict the associations between circRNAs and diseases, the Deep Auto-encoder (DAEN) algorithm is used by it to extract features and thereafter the Random Forest (RF) classifier is used to classify and predict the results of the score matrix. GCNMDA [38] is developed by Long et al. to predict the associations between human micro-organisms and drugs, with a Conditional Random Fields (CRF) layer added to the GCN process for feature extraction and final scores calculation. The SIMCCDA [39] is developed by Li et al. to predict the associations between circRNAs and diseases, which uses the PCA algorithm for feature extraction and dimensionality reduction, after which the Speedup Inductive Matrix Completion (SIMC) algorithm is used by it to perform the calculation of the prediction score matrix. The VGAELDA [40] integrates variational inference and graph autoencoders for lncRNA-disease associations prediction. The GATMDA [41] using graph attention networks with inductive matrix completion for human microbe-disease associations prediction. After fivefold CV, the AUC values of GCMDR, AE-RF, GCNMDA, SIMCCDA, VGAELDA, GATMDA and CRPGCN are 0.6882, 0.8653, 0.7714, 0.8291, 0.5114, 0.9254, 0.9720, respectively. The AUPR values are 0.1203, 0.8062, 0.1465, 0.1756, 0.6367, 0.9067, 0.9418, respectively. In addition, the results of performance evaluation indicators such as F1, MCC, ACC and RECALL are shown in Fig. 6 and Table 4. This study effectively combines circRNA sequence informations, circRNA gene informations, and disease DAG data by fusing multiple datasets. Thereafter, the RWR algorithm is used by CRPGCN for comprehensive similarity calculation, which allows each node being calculated to better fuse informations from neighbouring nodes with higher weights. PCA is then used for feature extraction and dimensionality reduction, and the similarity informations of the nodes is further enhanced. It allows each pair of circRNA-disease nodes with high similarity to perform more prominent features while also performing data noise reduction, so that the pre-processed datas can be used by the GCN for faster feature learning and to obtain a higher accuracy scores prediction matrix.

In summary, the CRPGCN algorithm has a higher accuracy and greater advantage in predicting the associations between circRNAs and diseases than many other excellent comparative algorithms.

### Comparison with different datasets

In order to verify the reliability of the CRPGCN algorithm under different datasets, this experiment provides 4 types datasets for comparison, as shown in Table 2. DataSet-1 has 330 types of circRNAs and 354 types of associations with 48 diseases; DataSet-2 has 661 types of circRNAs and 736 types of associations with 100 diseases; DataSet-3 has 512 types of circRNAs and 609 types of associations with 71 diseases; DataSet-4 has 533 types of circRNAs and 612 types of associations with 89 diseases. DataSet-4 is the benchmark dataset for this study.

**Fig. 8:**
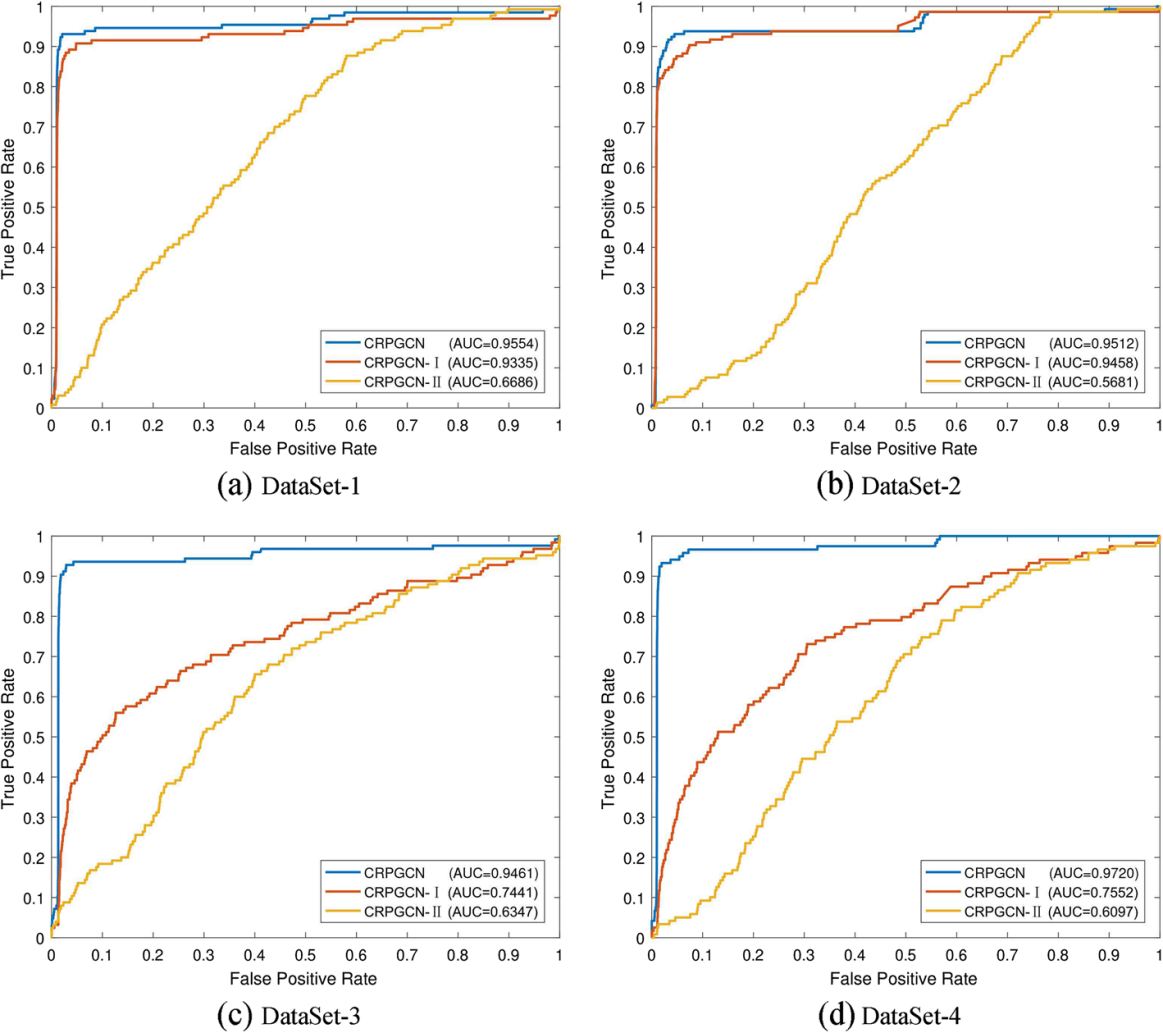
Fivefold cross-validation ROC curve compare with CRPGCN, CRPGCN-I and CRPGCN-II under different datasets

**Table 5:** Compare the AUC values of CRPGCN with different datasets under k-fold CV

DATASET	Twofold CV	Fivefold CV	Tenfold CV
DataSet-1	0.9263	0.9554	0.9498
DataSet-2	0.9407	0.9512	0.9653
DataSet-3	0.9392	0.9461	0.9611
DataSet-4	0.9490	0.9720	0.9722
Mean	0.9375	0.9562	0.9621

The histogram of AUC values in Fig. 7a. and Table 3 shows that the AUC values of the CRPGCN method under fivefold CV are consistently stable at around 0.95, with little fluctuation. Whereas CRPGCN-I also performs well on the DataSet-1 and DataSet-2, the AUC values produce a significant drop on the DataSet-3 and DataSet-4, indicating that for different datasets the CRPGCN-I method produces large fluctuations in its effectiveness, which implies that the CRPGCN-I algorithm is not stable. For CRPGCN-II, the results in the figure show that it performs relatively poorly in all four datasets, which implies that CRPGCN-II basically fails to make accurate predictions. The AUC values of CRPGCN algorithm for twofold, fivefold and tenfold CV in the four datasets are shown in Table 5, while the average AUC values of them are calculated and they are 0.9375, 0.9562 and 0.9621 respectively. In summary, it can be shown that the CRPGCN algorithm has the same stable, efficient and accurate prediction both under different datasets and in comparison with other computational methods.

**Fig. 9:**
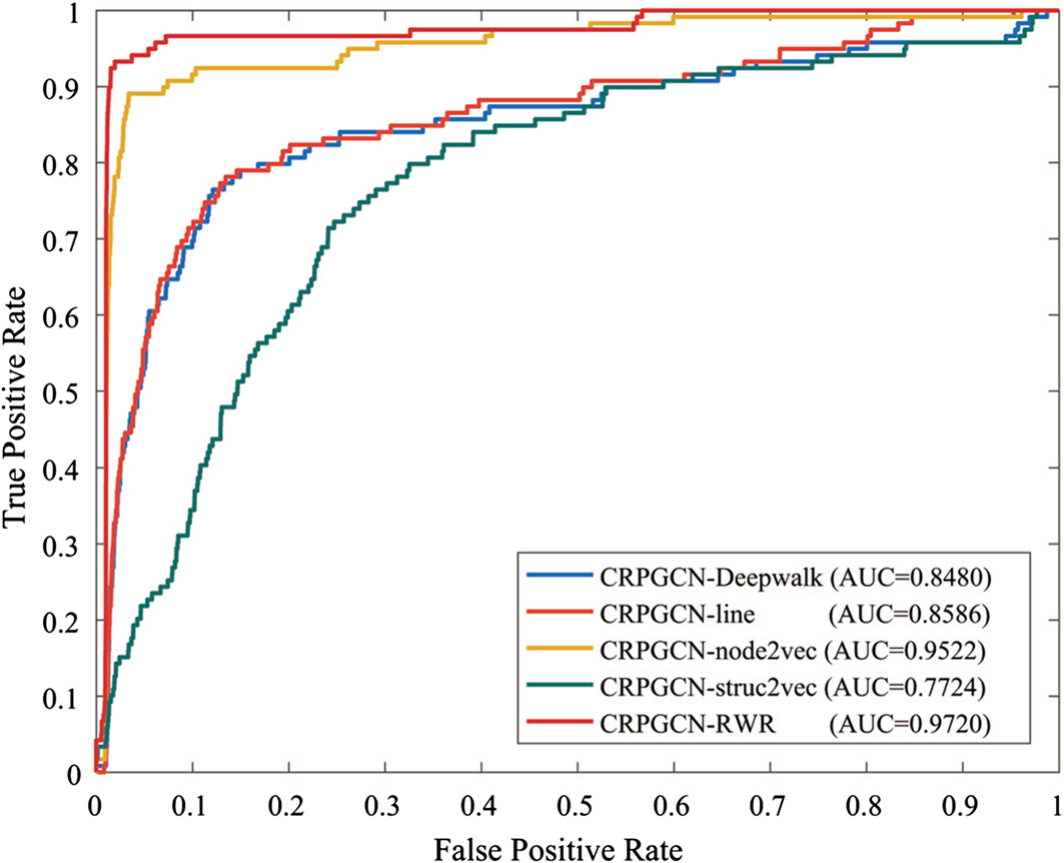
Compare the ROC curves with different calculation methods models under fivefold cross-validation

The four ROC curves in Fig. 8 show that the ROC curves of the CRPGCN algorithm all rise rapidly, with the TPR reaching above 0.9 before the FPR value of 0.1, which indicates that the CRPGCN algorithm is extremely efficient. For the CRPGCN-I method, the ROC curves under DataSet-1 and DataSet-2 are also rise fast, with TPR values reaching around 0.9 before the FPR value of 0.1. However, the curves of CRPGCN-I under the DataSet-3 and DataSet-4 are significantly flatter, with TPR values basically reaching 0.9 after the FPR value of 0.9. This performance indicates that for different datasets, the prediction accuracy of CRPGCN-I fluctuates somewhat. For the CRPGCN-II method, the curve trend is remarkably flat for either of the four datasets, along with low AUC values, which indicates that the CRPGCN-II method basically does not have accurate predictions for the associations between circRNAs and diseases. Furthermore, because of the inclusion of the PCA algorithm for extraction feature, the CRPGCN algorithm and the CRPGCN-I algorithm had higher AUC values than CRPGCN-II, which suggests that the PCA feature extraction algorithm is equally essential for this experiment. Meanwhile, although Dataset-4 is not the dataset with the most circRNA-disease associations, CRPGCN obtained the highest AUC value because this algorithm incorporates gene-based circRNA similarity for circRNAs composite similarity calculation, which shows that gene-based circRNA similarity is crucial for this algorithm.

### Comparison with different comprehensive similarity calculation method

In order to study the influence of different similarity calculation methods on CRPGCN algorithm, in addition to RWR, DeepWalk [42], Line [43], Node2vec [44] and Struct2vec [45] algorithms are selected for comparison.As shown in the Fig. 9, the AUC values of CRPGCN using RWR, DeepWalk, Line, Node2vec and Struct2vec similarity calculation methods reached 0.9720, 0.8480, 0.8586, 0.9522 and 0.7724 under fivefold CV respectively. This means that the RWR algorithm has better performance compared to other similarity calculation methods in this study.

In the data pre-processing stage, the comprehensive similarity between circRNAs and the comprehensive similarity between diseases is calculated for feature learning. However, simply calculating the comprehensive similarity is not sufficient to fuse the datas between similar nodes for feature learning, so it is necessary to fuse the neighbouring nodes based on the comprehensive similarity to help the subsequent feature extraction. Compared to the other similarity calculation algorithms in this study, the RWR algorithm focuses more on the influence of the weights of neighbouring nodes on the similarity calculation, and it uses the comprehensive similarity as the similarity weights of neighbouring nodes for datas fusion. In contrast, Struct2vec focuses more on the calculation of structural similarity, which does not have much influence on this experiment, so the AUC value of Struct2vec is the lowest. On the other hand, Node2vec is closer to the RWR algorithm in terms of computational results because it is also more concerned with the weights of neighbouring nodes. However, compared to the RWR algorithm, Node2vec uses either a Depth-First-Search (DFS) strategy or a Breadth-First-Search (BFS) strategy to calculate similarity which combines more informations from low similarity nodes, whereas the RWR algorithm may return to the original nodes for similarity calculation which allows the neighbouring nodes with high similarity to be combined more closely. Overall, the RWR algorithm is the best choice for the computation of similarity in this study.

### Case study

To further validate the predictive performance of CRPGCN for diseases, the case study is conducted on breast cancer alone. Breast cancer is a common disease and is one of the more lethal diseases especially for women. This case study may allow researchers to better study breast cancer and develop drugs or methods for effective treatment. The circR2Disease database and circFunBase [46] database are selected for validation. By removing circRNAs associated with breast cancer and then training them using CRPGCN, the final experiment predicted the unassociated data. The top 40 circRNAs are confirmed in descending order of prediction scores according to the CRPGCN method, as shown in Table 6 (see Additional file 6). There are some unidentified associations between circRNAs and breast cancer that may be able to be validate in future studies. The experimental results demonstrate the excellent predictive performance of the CRPGCN algorithm.

**Table 6:** Prediction of the top 40 predicted circRNAs associated with Breast cancer

Rank	circRNA	Evidence (PMID)
1	circBCL11B	29221160
2	hsa_circ_0108942	29045858
3	hsa_circ_0001875	28484086
4	hsa_circ_0001982	28933584
5	hsa_circ_0000893	28744405
6	hsa_circ_0001667	28803498
7	hsa_circ_0006054	28484086
8	hsa_circ_0003838	28803498
9	hsa_circ_0002874	28803498
10	hsa_circ_0001721	28744405
11	circDENND4C	28739726
12	hsa_circ_0000732	28744405
13	hsa_circ_0092276	28803498
14	hsa_circ_0068033	29045858
15	hsa_circ_0085495	28803498
16	MCF7_circ_000595	27829232
17	circBRIP	29221160
18	hsa_circ_0001824	28484086
19	circVRK1	29221160
20	circMED13	29221160
21	circOLA	29221160
22	hsa_circ_0008945	28744405
23	hsa_circ_0004214	28622299
24	hsa_circ_0008717	28744405
25	hsa_circ_0001283	28744405
26	hsa_circ_0004619	28484086
27	hsa_circ_0000981	28744405
28	hsa_circ_0001785	29045858
29	hsa_circ_0006528	28803498
30	hsa_circ_0093859	29593432
31	hsa_circ_0000098	28744405
32	hsa_circ_0004771	Unconfirmed
33	hsa_circ_0000911	28744405
34	circETFA	29221160
35	hsa_circ_0086241	28803498
36	hsa_circ_0091702	Unconfirmed
37	hsa_circ_0011946	29593432
38	hsa_circ_0008305	Unconfirmed
39	hsa_circ_0080210	Unconfirmed
40	hsa_circ_0041946	Unconfirmed

## Conclusions and discussion

In this paper, CRPGCN is proposed for predicting the relationships between circRNAs and diseases using GCN constructed with RWR and PCA based on heterogeneous network. In CRPGCN, data from multiple datasets are used for similarity fusion, which includes information on circRNA sequences, genes, DAG of diseases, and circRNA-disease associations . By filtering the dataset, 533 circRNAs with 89 diseases are obtained.

With above information provided by the datasets, the circRNA GIP kernel similarity matrix CIS, the sequence-based circRNA similarity matrix CES, the gene-based circRNA similarity matrix CGS, the disease GIP kernel similarity matrix DIS, and the disease semantic similarity matrix DSS are calculated. After that, the circRNA comprehensive similarity matrix CS is obtained by fusing CIS, CGS and CES, and the disease comprehensive similarity matrix DS is obtained by the fusion of DIS and DSS. Thereafter, the RWR algorithm is used to allow each node to learn the information of neighbouring nodes with higher correlation. However, the simple splicing matrix inevitably generates noise, and the PCA method not only enables feature extraction but also noise reduction for the splicing matrix. The datas processed by these methods are fused into a heterogeneous adjacency matrix and a heterogeneous feature matrix, which are used by the GCN algorithm for feature learning and calculation of associations scores between circRNAs and diseases. The results and comparative experiments show that the CRPGCN algorithm proposed in this paper has good performance and can accurately predict the associations between circRNAs and diseases. It can provide useful help to biologists and save their time in experiments.

Also, in the comparison experiments of this paper, the CRPGCN method has an outstanding performance in comparison with the best published algorithms. The results show that the CRPGCN method is the best among the comparative methods in this paper. In order to demonstrate the stability of the CRPGCN method, different datasets are used for the comparison. In conclusion, the different comparison experiments show that the CRPGCN algorithm is a stable and accurate prediction performance for the associations between circRNAs and diseases.

## Supplementary Information

**Additional file 1**: Adjacency matrix A. The adjacency matrix A constructed from circR2Disease.

**Additional file 2**: circRNA comprehensive similarity matrix.

**Additional file 3**: Disease comprehensive similarity.

**Additional file 4**: circRNA feature matrix.

**Additional file 5**: Disease feature matrix.

**Additional file 6**: Prediction of the top 40 predicted circRNAs associated with Breast cancer.

## Data Availability

The dataset and source code can be obtained from https://github.com/KajiMaCN/CRPGCN/. The circBase database can be downloaded from http://bioinfo.snnu.edu.cn/CircR2Disease/. The circR2Disease database can be got from http://circrna.org/. The MeSH database can be obtained from https://www.nlm.nih.gov/.

## Abbreviations

circRNA: Circular RNA
GCN: Graph convolutional network
RWR: Random walk with restart
PCA: Principal component analysis
CGR: Chaos game representation
DAGs: Directed acyclic graphs
TPR: True positive rate
FPR: False positive rate
ROC: Receiver operating characteristic
AUC: Areas under ROC curve
